# Structuring Formative Feedback in an Online Graphics Design Course in BME

**DOI:** 10.1007/s43683-021-00046-z

**Published:** 2021-02-12

**Authors:** Rucha Joshi, Sujoy Ghosh, Alexander Simileysky, Mayank Bhanot

**Affiliations:** 1 Department of Biomedical Engineering, University of California, 2318 GBSF, 451 Health Sciences Dr., Davis, CA 95616 USA; 2 Department of Materials Science and Engineering, University of California, Davis, CA 95616 USA; 3 Department of Electrical and Computer Engineering, University of California, Davis, CA 95616 USA

**Keywords:** Online instruction, Feedback, Formative, Computer aided design, CAD, Graphics communication

## Abstract

With a motivation to immerse students in engineering design, graphics communication, and computer aided design (CAD) skills early-on in the biomedical engineering curriculum, we launched a new 2-unit laboratory course on “Graphics Design in BME” in the Spring 2020 quarter for UC Davis sophomores. Due to the COVID-19 pandemic, the course met with the significant challenge of conversion to an online mode of teaching, instead of planned face-to-face instruction. Providing formative feedback was thought to be an important step to help students succeed in their final CAD project of the course. In the process of designing feedback, we found that the concept of feedback is still fragile in an online learning environment because online learning settings provide distinct pedagogical demands as compared to face-to-face settings. The situation is especially delicate in the context of contemporary higher education imparting engineering skills, where students attend large classes, with diminished opportunities to interact with the teaching staff. The challenge we faced was to provide meaningful dialogic feedback in an online environment, especially while teaching engineering graphics design to a large class. Here we addressed this challenge by focusing on the process of structuring meaningful feedback. We designed a project assignment to be submitted as multiple deliverables to be submitted in two-stages. Then, we characterized its feedback with multiple notions, such as dialogic iterative cycles, personalized, goal-directed, immediate, in written format, and having a peer assessment component. The process of providing formative feedback online through the structure mentioned in this paper resulted in students’ improved scores on the final project elements. It also helped us identify the common issues students are faltering in a graphics design class, and provide customized guidance, ideal examples of expected work, and more resources to motivate each student group to achieve mastery of course content.

**Supplementary Information:**

The online version contains supplementary material available at 10.1007/s43683-021-00046-z.

## Challenge Statement

The COVID-19 pandemic-based shift of decision to launch our new course on Graphics Design in BME in an online mode instead of face-to-face, raised a big challenge in terms of providing meaningful feedback to a large, online class of students for improving their problem solving, graphics communication, and design skills. Feedback was needed for reducing discrepancies between students’ understandings and the goals of the assignment.^15^ However, the conditions that make feedback possible in face-to-face instruction, such as observation of students’ work over time and multiple opportunities to provide useful comments, are diminished in an online course. Compounding the problem further are factors such as larger class size and a lack of students’ training in how to interpret and utilize feedback for improving performance on task.^3^,^23^

Many theoretical models describe the mechanism of feedback production, how it is received, and what effect it has on students.^3^,^4^,^8^,^18^ These models have been used to formulate principles to provide feedback,^6^,^20^ including that of sustainable feedback practice.^5^ Despite the volume and rigor of studies investigating feedback, we found that the concept is still fragile^9^ and inherently problematic.^16^ Literature indicated that the feedback process needs to be made more dialogical and ongoing through discussions, clarifications, and negotiations between student and tutor.^5^ However, this dialogical nature of feedback requires a fair deal of interactivity between student and teacher, which is inherently difficult to achieve in an online environment. Opportunities for informal engagement outside the classroom, such as discussions with other classmates, tutors, *etc*. are also reduced in an online environment, which would otherwise help and equip students with a better appreciation of what is expected of them, and develop their understandings of academic terms and appropriate practices as they begin to write project reports. This situation is especially delicate in the context of contemporary higher education imparting engineering skills, where students attend large classes with fewer opportunities for an individual student to interact with teaching staff.

## Novel Initiative

Most works in the past have treated feedback as a singular concept. Here we treated feedback as more than one notion and focused primarily on the needs of learning rather than the capacities of the teacher as suggested by Boud and Molly.^3^ We focused on the feedback process, format, personalization, content, function, and presentation as described in Sect. 2.1, and implemented it online. The salient features of our feedback were: its written and dialogic format, personalization for each team, and facilitation of two-stage assignment submission. Assessment of impact caused by our formative feedback as presented in this paper showed there was an effect in the desired direction.

The aim of this paper is to: (1) report how we structured formative feedback in an online transitioned course on Graphics Design in BME, in response to the COVID-19 pandemic, and (2) report the assessed effects of the formative feedback provided online, to enhance students’ performance in a computer-aided bioreactor-design project.

### Learning Objectives for the Course and Student Project

The Learning Objectives (LOs) of this course were set such that at the end of the course, students would be able to:

1. Apply spatial visualization and create orthographic and isometric views of objects.
2. Construct 2D and 3D drawings, and assembly and sub-assembly structures.
3. Follow engineering design procedures including problem identification, problem formulation, approaches, methodology, and solution.
4. Use industry-standard software packages for the design of a 3D Computer-Aided Design prototype of a bioreactor.
5. Work in a team to document and present the process of Computer-Aided Design of their product.
6. Develop an ability to utilize online learning tools and collaborate online.

Specifically, an end-of-the-quarter bioreactor project was designed to teach students to (i) identify a biomedical graphics design challenge for creating a bioreactor that satisfies a given need, (ii) evaluate various parameters for the optimal design of the bioreactor based on literature search, (iii) Select specific parameters for bioreactor design using a weighted decision matrix, (iv) synthesize a preliminary concept of the prototype on paper and in CAD, and (v) communicate the model formation process and drawing in CAD accurately in the form a written report.

All students had a common challenge for their project—to create a bioreactor using CAD for enhancing the viability and proliferation of mesenchymal stem cells on porous silk-fibrin 3D scaffolds (project details are in Supplementary Information). The course had 98 students split into 24 teams. Each team designed a bioreactor using CAD. The project constituted 15% of the final grade, including a written project report (10%) and a CAD portfolio (5%).

The project was divided into several assignments due week 2 to 9. Final projects were due in week 10 of instruction. Rubrics and additional resources were provided to students for each assignment. Written formative feedback was given using Canvas LMS through weeks 2–6. We analyzed the pre and post-feedback performance of students after coding the data upon approval of the Institutional Review Board (IRB). The timeline of project deliverables is indicated in Table S1. The Project Need Statement, Goals & Objective, Decision Metrics, and Preliminary Concept were assessed by the instructor. Hand-drawn Sketches were assessed *via* a peer review process, where at least three different students graded a submission anonymously, and scores were averaged by a TA.

### Structuring Feedback

Research shows that viewing feedback more as dialogue than the transmission of information,^20^ and providing high-quality formative feedback in iterative cycles^1^ while assuring that students engage with it, facilitates and promotes learning.^2^ However, complex feedback could have negative effects on student motivation,^18^ so we carefully designed our feedback to be a continuous intervention, as described below.

*Dialogic feedback* suggests an interactive exchange in which interpretations are shared, meanings are negotiated and expectations are clarified.^5^ Dialogic feedback is impacted by *iterative cycles* such as two‐stage assignments or multi-stage assignments that can involve feedback on the first stage, intended to enable the student to improve the quality of work for a second (or later) stage submission.^12^ To facilitate iteration, we divided the students’ Computer-Aided Design (CAD) project into several project elements (table S.1 in Supplementary Information), each tasked into a two-stage assignment, initial and final, both graded by the same grader. Such iterative two-stage or multi-stage assignments are shown to be more promising than the end of the quarter assignments or examinations and have been reported to facilitate sustainable and dialogic feedback.^5^

**Figure 1:**
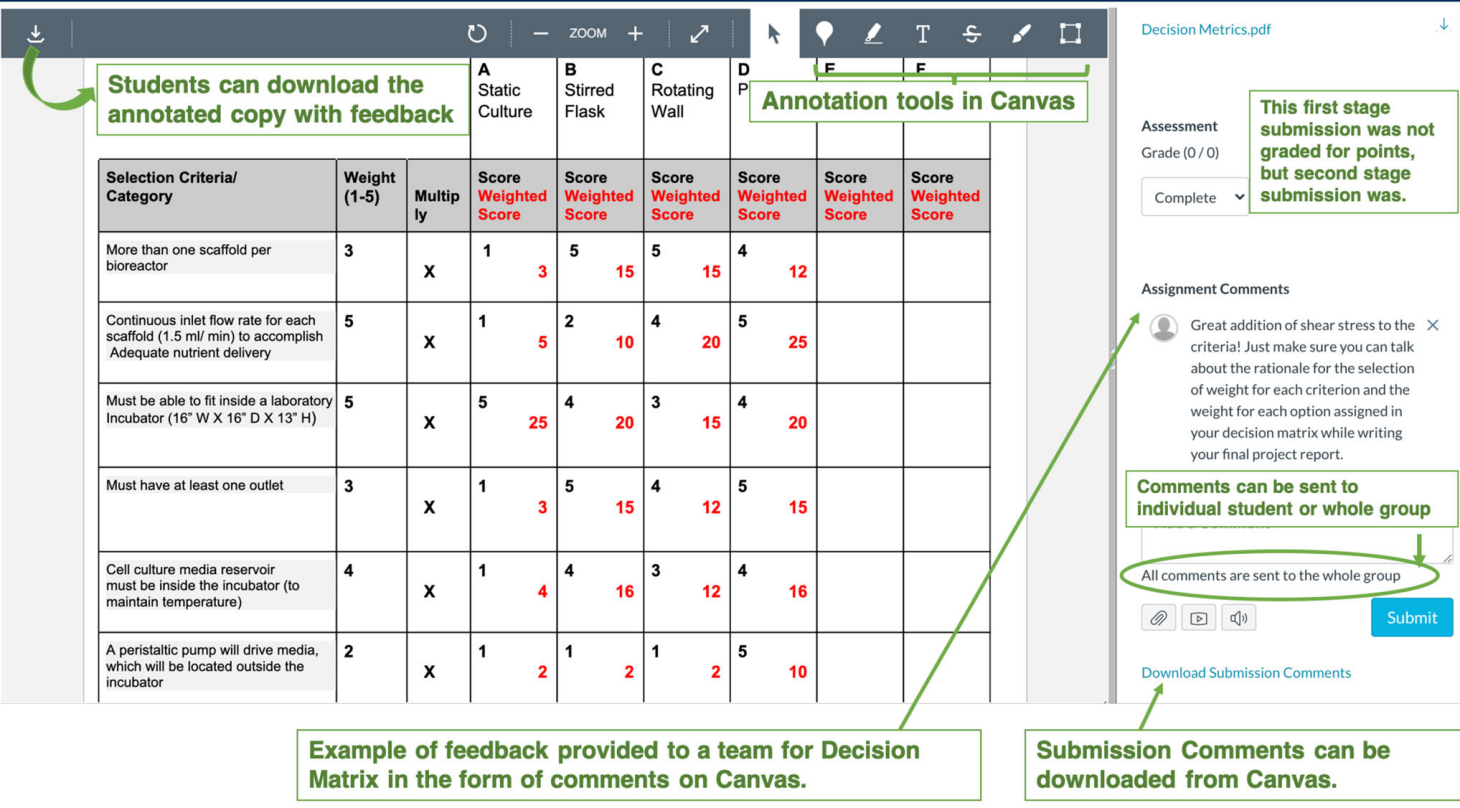
Example of feedback provided to a student team through Assignment Comments function on Canvas.

**Figure 2:**
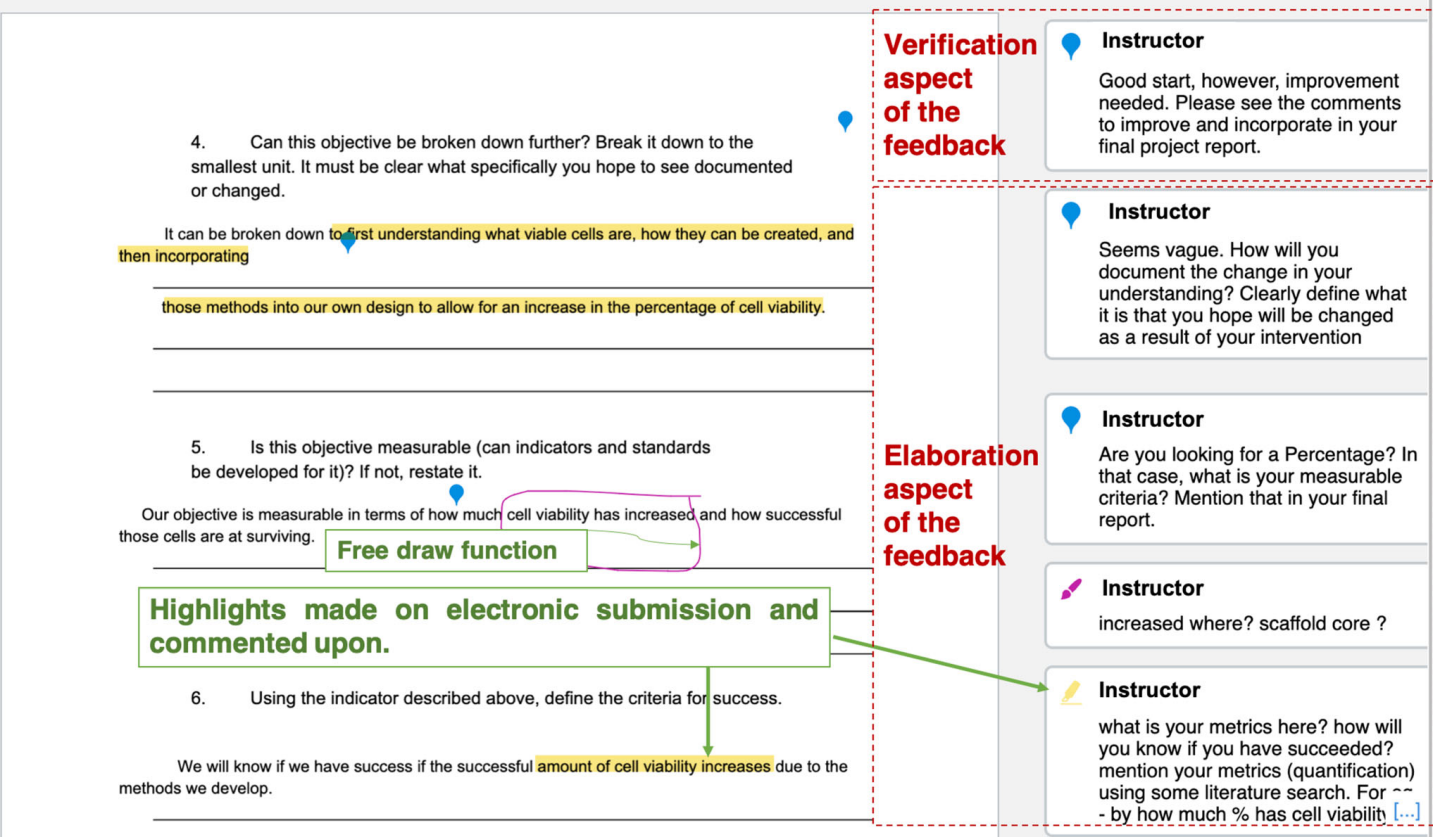
Examples of feedback provided to student teams for the Goals and Objective assignment. Personalized feedback shows verification and elaboration aspects.

Personalization of the feedback allowed tailoring comments to students’ learning needs.^7^ Personalization was operationalized at two levels: (1) mistakes in assignments were identified and communicated to teams, (2) information tailored to each student’s needs was reported to that individual student in the team using the Canvas Learning Management system (LMS) as shown in Figs. 1 and 2. It is known that the more personalized feedback is, the more dialogue it will support.^7^

Another element that influences the potential for dialogic feedback is *peer feedback.* Peer feedback involves students engaging in reflective criticism of other students’ products.^7^ We designed one of the feedbacks in our course assessment as peer feedback (element 2 c—hand-drawn sketch of the bioreactor). Since student expectations and objectives can differ from the instructor’s objectives, a peer evaluation rubric was given to all classmates, as shown in Supplementary Table S4 for Week 6 assignment. We facilitated the provision of anonymous peer feedback using Canvas LMS.

Feedback *content* is also important, where ‘content’ involves both the verification and elaboration aspects. The verification is a simple judgment of whether an answer is correct, and elaboration provides hints, cues, analogies, explanations, *etc*.^24^ (e.g., see comments in Fig. 2). We also used a scaffolded, directive feedback approach where students were encouraged to reach the next goal of the assignment by addressing the points mentioned in the first feedback. Our feedback *function* was therefore directive in nature.

Finally, the *presentation* of our feedback components was *immediate*; provided within 1–2 weeks of students’ assignment submissions (see Supplementary Table 1). This decision was based on Mason and Bruning’s framework for computer-based feedback.^19^

We provided feedback mainly in written format than oral format, because Chickering and Ehrmann’s^6^ essay suggests that it is often easier to discuss values and personal concerns in writing than orally since inadvertent or ambiguous nonverbal signals are not so dominant in written format. Thus we had an opportunity to converse and exchange work through Canvas LMS more thoughtfully and “safely”.

### Examples of Feedback in Canvas

We used the *Comment* feature and the *Highlight and Free Draw* annotation types in the *point annotations* as shown in Canvas DocView in Figs. 1 and 2 to give feedback. Other annotation features such as the *Free Text, Strikeout* annotation types were also found useful. Students could view our DocView comments from the assignment Submission Details page. The Assignment Comments section (Fig. 1) allowed for written dialogue. We could also send a comment to an individual student in the team if required, or to the whole group by selecting ‘Send comment to this student only’, or ‘Send comment to the whole group’ option respectively (Fig. 1).

## Reflection

Sadler^23^ emphasizes feedback may simply be viewed as ‘dangling data’ if strategies are not provided for improving learning and monitoring how performance information subsequently influences the learner. We avoided making feedback such a ‘dangling data’ for students, by structuring project assignments as a two‐stage submission and provided rubrics, resources to students to improve their work before resubmission. Moreover, breaking the final project into various tasks to be completed weekly agreed with the important principle that assessment should inspire an even distribution of study time throughout a module, rather than being concentrated at its end.^11^

**Figure 3:**
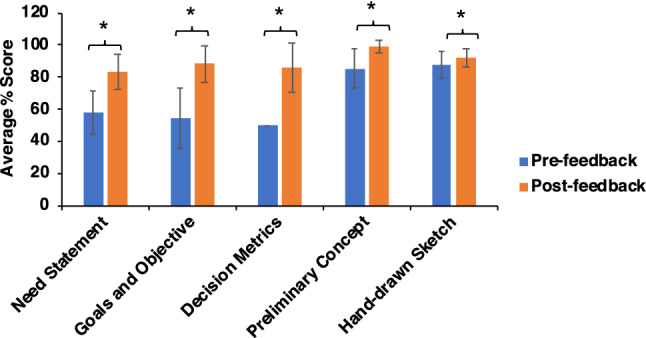
Impact of feedback on students’ scores of project elements before and after receiving the feedback: Each element was assessed twice, before feedback and after feedback. The total number of student teams was 24. Asterisk marks (*) indicate statistically different experimental groups as determined by a paired two-sample test performed on each element’s pre-feedback and post-feedback scores for 24 teams. (*N* = 24, *p* < 0.05).

The structuring of all student project deliverables into two-stages also allowed us to detect feedback effect on online learning.^22^ Assessing the performance of students on elements 1a, and 1b, and 2 a, 2b, 2c (Fig. 3), we found that there was an increase in students’ final scores compared to their initial scores on each assessed project element. We believe this increase in student performance can be attributed to the personalized nature of feedback, that emphasized the connection with learning and promoted higher student engagement, in agreement with the results from literature suggesting the use of such strategies in face-to-face instruction.^3^,^21^ The median student grade on final CAD projects was 96%, the lowest was 89.33%, and the highest was 98.33%.

We analyzed pre and post-feedback assignments for all project elements except the last two project elements-3 (CAD Method) and 4 (Drawings using Solidworks) because some teams were still working upon their CAD parts at the time of feedback (week 9). To facilitate feedback provision on these deliverables in the future, we will shift the last two deliverables by a week earlier.

One project element—the Hand-Drawn Sketch warrants special discussion. The hand drawn-sketch assignment required anonymous peer feedback which was enabled *via* Canvas LMS. Detailed rubrics were provided to every student and each submission was analyzed per rubric by three students anonymously. We observed that the peer feedback process enhanced student engagement with the course content. Several students provided elaborate feedback with specific comments about positive and negative issues in a submission. Our experiences about peer feedback processes are in agreement with previous findings showing that the opportunities for dialogic peer formative feedback promote learning support and self-regulation.^13^

While the feedback process significantly enhanced the students’ final scores in all project deliverables, not all student teams reached an ‘Accomplished’ and above (Exemplary) level in the rubric, which essentially meant scoring more than 80% on a project deliverable. This is illustrated through data in Table 1. Performance of teams on the assignments—Need Statement, Goals and Objectives, and Decision Matrix—indicated the weakness in applying the engineering design principles, which the students had studied in their first year. In contrast to these engineering design based assignments, the Preliminary Concept and Hand Drawn Sketch assignments showed higher class performance level. To address this discrepancy, we will place greater emphasis on engineering design principles in teaching the course in the future. However the increased achievement in graphics design assignments could be attributed to some other factors such as the related class content being fresh in students’ minds; assignment being closer to the submission of the final report, better global picture of the project and role of this assignment in the project; a visual learning process happening *via* graphics/drawings; better interpretation of feedback by students than on the earlier assignments. We did not do any follow-up interviews or student surveys on this issue, but this will be worth doing in the future to know the underlying causes.

**Table 1:** Number of teams that reached the Accomplished or Exemplary category.

Assignment	No. of teams out of 24 scoring > 80% marks	Class percentage
1. Need statement	16	66.67
2. Goals and objective	22	91.67
3. Decision metrics	21	87.50
4. Preliminary concept	24	100.00
5. Hand-drawn sketch	23	95.84

Reflecting deeper on the student performances in the first three assignments of the project, namely the Need Statement, the Goals and Objectives, and the Decision Metrics assignments, we realized a need to introduce another competency in this course and train students to interpret the given feedback. Studies such as that by Winstone^25^ have revealed that many barriers can inhibit the use of feedback, ranging from students’ difficulties with decoding terminology, to their unwillingness to expend effort. Whereas the barriers identified could all in principle be removed, doing so would typically require a sharing of responsibility between teacher and student. This highlights the importance of training students to be proactive receivers of feedback, especially in an online environment. By structuring learning environments supportive of students’ metacognitive training,^20^ we can train our students to self-monitor their project progress and interpret and apply available feedback efficiently for improving the quality of their final product.

**Figure 4:**
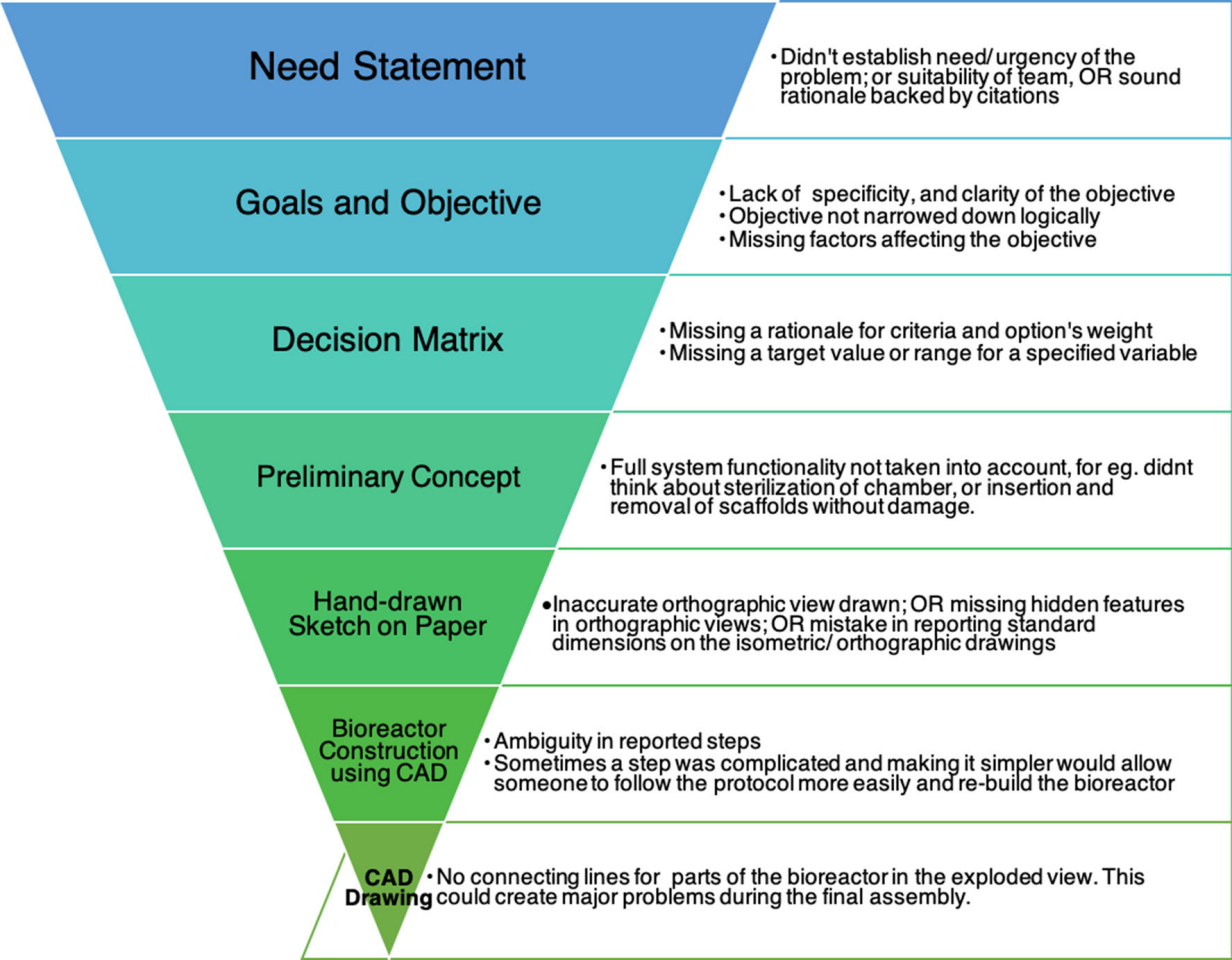
Project deliverables arranged in an inverted pyramid, narrowing down to the final task of Computer-Aided Design and Drawing of a bioreactor. Mentioned on the right-hand side are the common shortfalls observed in each type of deliverable submitted by student teams.

The assessment of student project deliverables while constructing feedback also helped us spot the common issues that students were faltering upon in each project deliverable. These ‘common issues’ were mistakes found in submissions of about 5–6 teams (about 1/4th of a class) in their first iteration of the project deliverables. The early review of student-work helped us find these shortfalls, as listed in Fig. 4. Spotting these mistakes was particularly helpful in the online environment because informal discussions and places for ongoing monitoring of evidence of learning are difficult to attain online.^14^ Identification of any common mistakes we came across in the assessment of the first iteration of project deliverables, helped us to provide concrete, directive, and personalized feedback to teams for improvement while alerting the class to avoid such issues in the final submission. More importantly, identifying common errors helped us to adjust instruction to clear misconceptions about specific content/topics before students submitted their next iteration of the assignment.

**Figure 5:**
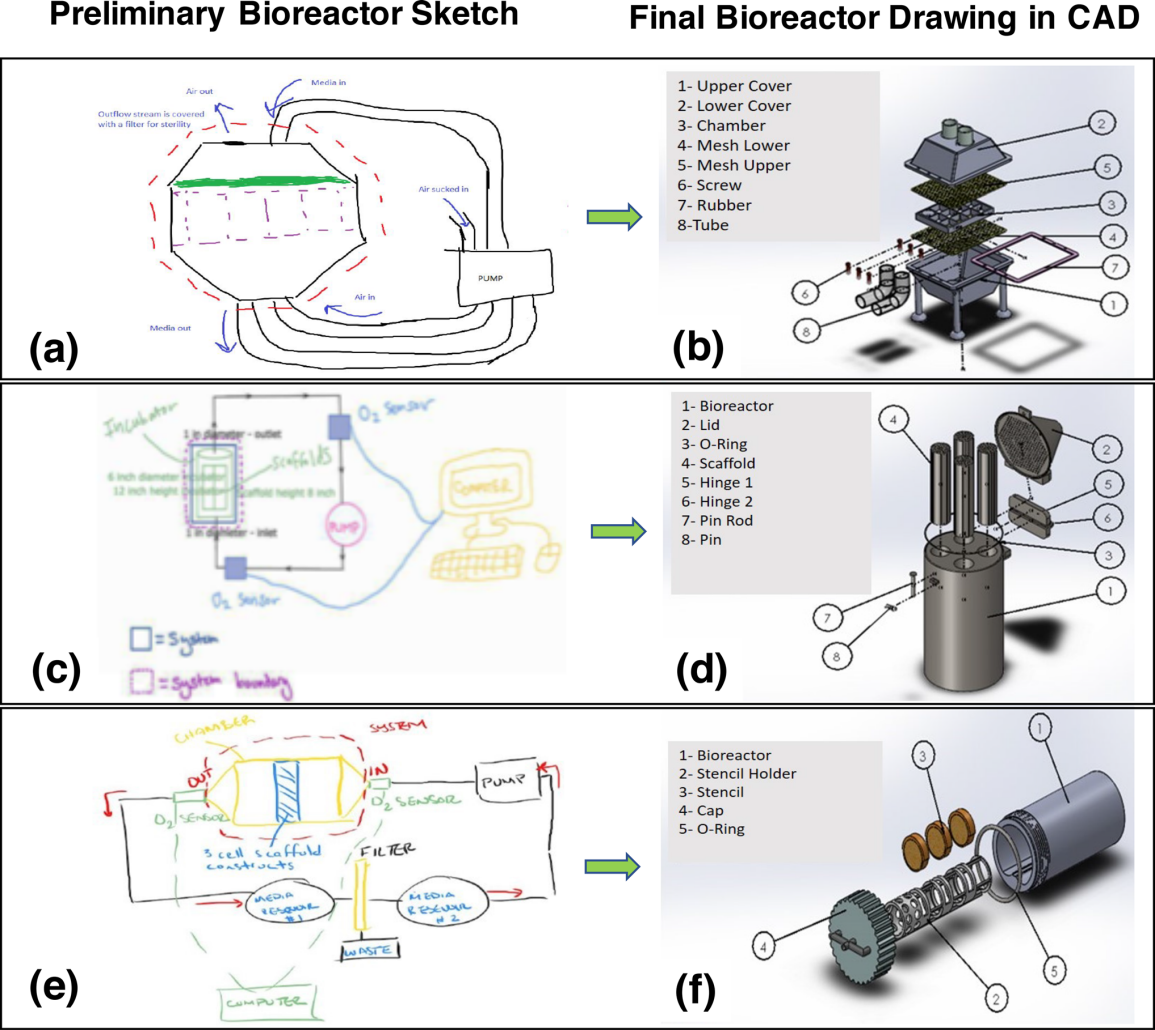
Representative images from student submissions for project deliverables showing the evolution of drawings from simplified preliminary concept drawings (a, c, and e) to the final bioreactor CAD designs (b, d, and f). Drawings shown on the left-hand side of the arrows were created by student teams on paper, and drawings shown on the right-hand side of the arrow were created by the same teams in Solidworks. The final bioreactor models in CAD represent the multilayer series assembled reactor with easy to add/remove components, suitable for continuous flow of culture solution (part b); a cylindrical bioreactor with quick installation of scaffolds in hinge type chamber suitable for a heavy-duty operation involving high fluid flow volumes (d); and a lightweight, hollow bioreactor with reduced material use for stencil design (f), which also increases the volume of culture solution flowing through the reactor.

Student’s progress in submissions showed progressive evolution of drawings from simplified preliminary concept drawings to more concrete, practical bioreactor CAD designs. For example, images in Figs. 5a, 5c, and 5e, indicate the different teams’ preliminary concept sketches on paper that were later utilized to build their final bioreactor models (Figs. 5b, 5d, and 5f) using Solidworks. About 16% of teams (4 out of 24) even went beyond making the first iteration of their bioreactor in CAD, and after seeking feedback from teaching staff about it in week 9, drew an entirely new preliminary sketch on paper before re-building their new bioreactor model in CAD. Other teams focused on improving the quality of their CAD model sticking to their idea of a preliminary sketch. For e.g., connecting lines that are seen in the exploded view of CAD in Figs. 5b, 5d, and 5f, were initially missing in some teams’ submissions, that were improved upon after feedback.

The written mode gave us an excellent means to provide more intimate and protected feedback to student teams. The written form of feedback was perhaps less intimidating than the demands of face-to-face communication. This was illustrated by the fact that we received requests from 6 student teams (out of 24) for additional feedback on their iterations of the project, beyond the feedback cycle implemented that quarter. Furthermore, typed annotations of the student submissions eliminated any possible barrier of the illegibility of any written feedback. In Ferguson’s study,^9^ a large number of students reported difficulty reading written responses from teaching staff. This issue was eliminated by typed, easily accessible, and downloadable feedback in Canvas LMS.

One observation this quarter was that students, in general, lacked coordination efforts for the final assembly of their project and were not comfortable approaching teammates in difficulties since the pandemic began. In that vein, a teammate rating assignment and continuous feedback for improvement can be helpful, as shown in Supplementary Materials (S2). Another idea is to go over the team-work logistics with each team, such as how are they going to make decisions about process, leadership, deputization, and coordination.

## Conclusion

We identified feedback characterized by multiple notions, such as dialogic iterative cycles, personalized, goal-directed, immediate, in written format, and having a peer assessment component. The process of providing formative feedback online through the structure mentioned in this paper helped us identify the common issues students are faltering at in a graphics design class and gave us opportunities to provide customized feedback, and ideal examples of expected work. The process of giving feedback inspired us with ideas and resources to teach innovatively and help students to achieve mastery of content in this course. It also possibly provided opportunities for metacognition to students by having them reflect on their previous assignment iteration.

The formative feedback type we gave in the online environment resulted in students’ improved scores on the final project elements. These results agree with many benefits of providing formative feedback reported in the literature^10^,^17^,^24^ Continuing to offer high-quality formative feedback and improve upon such formative, dialogic, personalized, and written feedback will be important for us in the current COVID-19 pandemic, as well as future online instruction. Efforts will be made to make sure that students engage with the feedback given, and that the given feedback facilitates and promotes learning.

## Supplementary Information

Below is the link to the electronic supplementary material.

Supplementary material 1 (DOCX 51 kb)
