# Supplementary material for: Structuring Formative Feedback in an Online Graphics Design Course in BME
Source: Biomed Eng Educ. 2021 Feb 12;1(2):325–33. doi: 10.1007/s43683-021-00046-z (PMC7880523; doi:10.1007/s43683-021-00046-z)
Supplement: Supplementary file 1 — Supplementary material 1 (DOCX 51 kb) [file 43683_2021_46_MOESM1_ESM.docx]

**Supplementary Material**

**S.1 Student Project Details**

Students were given the following problem for their CAD project.

*You are a researcher in a tissue engineering laboratory trying to grow a 3D tissue engineered product composed of human mesenchymal stem cells embedded on a porous silk fibroin scaffold. When culturing your cells on the 3D scaffold in a standard cell culture plate, you notice that the cells at the center of this cylindrical scaffold (8mm diameter, 4mm height) are necrotic. To improve cell viability and enhance cell proliferation, you would like to design a bioreactor to ensure that cell culture media is reaching all the cells in the scaffold.*

Then, the students were encouraged with following questions for analyzing the given problem in more depth, while guiding them to appropriate resources to find the answers systematically.

- What is tissue engineering? What is unclear to you in the problem statement?
- What is the advantage of culturing cells on a three-dimensional scaffold vs. monolayer?
- Why are cells necrotic at the center of the scaffold when cultured in a standard cell culture plate?
- What is a bioreactor?
- Why should you use it?
- What type of bioreactor should you use?

Following on the question about what a bioreactor does, we provided students with more information in lecture about bioreactor and some resources such as journal articles [1, 2] that discussed the different parameters to consider in a bioreactor for tissue engineering functionality. We also provided students with following URLs to **video protocols from JOVE** that showed the operation of a bioreactor in the context of tissue-engineering.

- <https://www.jove.com/science-education/5799>
- <https://www.jove.com/video/50460>
- <https://www.jove.com/video/50560>
- <https://www.jove.com/video/55322>

After giving student an opportunity to work on getting a clarity of the problem in week 2, we asked students to work through weeks 3-9 on specific tasks listed in Supporting Information:

- Understanding specific constraints that we provided them for designing a bioreactor
- Working on a decision matrix to arrive at a decision for making a particular type of bioreactor to address these and more constraints of their own fueled by literature search
- Presenting a preliminary concept of a bioreactor design on a paper
- Hand-drawing a sketch of that bioreactor in orthogonal and isometric views, following the principles of graphics communication learned in class
- Constructing the bioreactor in parts in SolidWorks and assembling it
- Presenting the final design drawings and documentation of the entire design process in the form of a written report

After allowing students to research online and get a clarity of the problem in week 2, we asked students to work through weeks 3-9 on specific tasks related to this project, as listed in Table S.1.

| **Supplementary Table S.1: Bioreactor project deliverables** | | | | |
| --- | --- | --- | --- | --- |
| **No.** | **Project element** | **Week** | **Guidelines / Resources provided to students** | **Grader** |
| 1 | Teammate expectation agreement  (Assignment 1) | 2 | - Getting to know you, Team policies and Team expectations agreement page given from [3]. - A [video on design thinking](https://www.youtube.com/watch?v=psLjEBUOnVs&feature=emb_logo) and a video question sheet provided [4]. | Instructor |
| 2 | Need Statement  (Assignment 2) | 3 | - Powerpoint Slides on dos and don’ts on need statement - How to develop strong need statement [5] with examples of good and bad statements. | Instructor |
| 3 | Goals and Objective worksheet  (Assignment 3) | 3 | - Excerpts from the 2010 User-Friendly Handbook for Project Evaluation [6] - Goals and Objective worksheet. | Instructor |
| 4 | Decision Matrix  (Assignment 4) | 4 | - Engineering Design decision matrix worksheet from [7]. | Instructor |
| 5 | Preliminary Concept  (Assignment 5) | 5 | - Directions given in lecture, system drawings taught in BIM 20 – fundamentals of Bioengineering class. | Instructor |
| 6 | Bioreactor Sketch (Assignment 6) | 6 | - Directions given in lecture - Peer review rubric given in Assignment 6. | Peer Review using Rubrics |
| 7 | CAD portfolio making & report writing | 9 | - Tutorials in lab and in SolidProfessor | TAs |
| 8 | Final project report due | 10 | - | Instructor + TAs |
| 9 | Teammate review required only for teams with a conflict  (Assignment 7) | 11 | - Virtual meeting with the TA of lab - Teammate review template with instructions | Instructor |

**S.2 Teammate feedback and improvement assignment**

(Developed based on model suggested by: Wilding, W. V., & Knotts, T. A., & Pitt, W. G., & Argyle, M. D. (2012, June), *Developing and Assessing Leadership in Engineering Students* Paper presented at 2012 ASEE Annual Conference & Exposition, San Antonio, Texas. https://peer.asee.org/21181)

By **noon,** **June 4^th^**, referring to the List of Leadership Qualities below, please provide to me for each member of your team a description of one or two strengths he or she possesses and one or two aspects of teamwork or leadership where improvements could be made. Write the name of individual you are providing feedback for in the header. These will be compiled with feedback from the other members of your team and distributed to the appropriate individual anonymously for their improvement.

**List of Leadership Qualities**

1. Exhibits high ethical standards.

2. Is reliable and can be counted on to accomplish tasks in a manner that exceeds expectations.

3. Takes initiative rather than waits for assignments.

4. Follows as well as leads.

5. Identifies problems and solutions.

6. Takes time to evaluate personal performance as a team member and improves when needed.

7. Receives criticism and makes changes where appropriate.

8. Demonstrates a good attitude on life and is pleasant to work with.

9. Is an effective communicator, including being a good listener.

10. Gives honest feedback to others and helps them succeed in their responsibilities.

11. Understands the personality traits of self and others and can work with others in accomplishing tasks.

12. Is culturally sensitive and works effectively with people from diverse backgrounds.

13. Develops vision in his/her scope of responsibility.

Once you have received the feedback from your team members, by **noon, June 9^th^**, submit (1) a brief summary of the feedback, (2) an evaluation of your own performance (including a reflection on your goals from your previous team project), and (3) a statement of your plan of how you will improve your teamwork and leadership abilities in your next team experience.

**S.3 Rubric**

Students were instructed to upload the **team's final project report** following to Canvas LMS. Following guidelines were provided.

| **Supplementary Table S.2: Project report estimated pages and guidelines** | | |
| --- | --- | --- |
| **Project Element** | **Pages** | **Points** |
| - 1. Introduction – understanding the challenge | 2 pages | 20 |
| - 1. Knowledge building – Rationalized decision-making and concept generation | 3 pages | 30 |
| - 1. Method of building bioreactor in CAD (tutorial expanded in Appendix) | 1 page | 10 |
| - 1. CAD-based Drawings | 4-5 pages | 20 |
| - 1. Reflection on project | 1 page | 10 |
| - 1. Writing quality | - | 10 |
| **Appendix** | **No limit enforcced** | **-** |
|  | **Total main report: 12-15 pages** | **100 points** |

**See rubric table below** for details of expected deliverable on each of the criteria.

Please place a cover page on the report that includes the team members’ names, student IDs and section number. Project report should be between **12-15 pages, single or double spaced, Arial font, size 11 or larger.**

**Note- When grading, the “Exemplary” mastery level (category) on each criterion will be referred before the other mastery levels** (i.e., Accomplished, Developing, Unsatisfactory) for evaluation. The Exemplary mastery level articulates the highest learning outcome.

Make sure to view instructor’s feedback on your individual lecture assignments for Project Elements 1 and 2 that you have already completed before iterating upon them for final project.

You will upload your final project reports as a pdf to Canvas.

| Supplementary Table S.3: Peer Assessment Rubric Criteria | | | | | |
| --- | --- | --- | --- | --- | --- |
| Criteria | **Sub-criteria** | **Exemplary (>9 points)** | **Accomplished (8-9 points)** | **Developing (4-7 points)** | **Unsatisfactory (1-3 points)** |
| 1. Intro-duction – Understanding the challenge | 1a. **Need Statement**  # Address Four questions –   - What is the need/problem? How do you know it is a need? - What is the target population that you are trying to help? - Is the problem/need urgent? Why does it need to be addressed now? - Why should it be your team that addresses the need? | - Describes why growing cells in 3D might be important for the target population - Addresses all questions of need statement with clarity and flow. - Lays out thoughtful solution to solve the problem - Provides a strong rationale for the solution, based on citations. - Provides citations | - Describes why growing cells in 3D might be important for the target population - Addresses all four questions of the need statement. - Lays out a solution to solve the problem. - Provides a rationale for the solution, based on citations. - Provides appropriate citations in text | - Describes why growing cells in 3D might be important for the target population - Addresses 3 of the four questions of the need statement. - Lays out a vague solution to solve the problem. - Provides a rationale for the solution. | - Addresses some of the four questions of the need statement. - Lays out a vague solution to solve the problem. |
|  | **1b. Goals and Objective** | - Goals and objectives are narrowed logically, are specific, clear, and provide a target comparison value. - Includes all factors that may affect the experimental results | - Goals and objectives are specific, clear and provide a target comparison value. - Includes most factors that may affect the results of the experiment. | - Goals and objectives are specific but miss target comparisons. - Includes only some factors affecting the experiment. | - Goals and objectives are vague, lack a target comparison and miss many factors that may affect the results of the experiment. |
| 2. Knowledge building - Rationalized decision-making and concept generation | **2 a. Decision Metrics** | - Identifies several possible criteria including those provided to the team and those that the team found on their own through literature. - Criteria / constraints show a range of target value. - A sound rationale is provided for each criteria weight and each option’s weight using citations. - Combines several investigations and research to learn about the problem, how the system works, relevant cases, and prior solutions. | - Identifies all critical criteria and constraints that relate to the goal of the design task. - Constraints are presented with target value. - Rationale for each criteria weight and each option’s weight is provided with some literature citation. - Evidence of using several given tools such as research papers, to learn about the problem, how the system works, relevant cases, and prior solutions. | - Includes only some criteria/ factors that may affect the results of the experiment. - Some rationale for criteria weight and option’s weight is provided. - Conducts minimal research before continuing the problem-solving process. | - Makes no active   attempt to identify and document critical criteria.   - Lacks explanation of factors that affect results. - Prematurely attempts to solve without identifying key components/criteria in problem structure. - Skips all research and begins solving. |
|  | **2 b. Preliminary Concept of Bioreactor** | - Bioreactor system is correctly identified, and labeled with boundary - System functionality is accounted for, such as sterilization, insertion and removal of scaffolds without damage, etc. | - Bioreactor system is correctly identified, and labeled with system boundary - Most of the system functionality is taken in account | - Bioreactor system is correctly identified, and labeled with system boundary - Some system functionality is taken in account | - Bioreactor system is incorrectly identified, and / or incorrectly labeled with system boundary - System functionality is not taken in account at all |
|  | **2 c. Hand drawn sketch* : View and feature- location, size and scaling**  *(*This element was anonymously peer assessed in first iteration, see* ***peer assessment rubric in Supplementary table S.3****).* | - Location, and scaling of Ortho-graphic views is accurate using Isometric Drawing - All Hidden features in Orthographic views are represented correctly - All Standard Dimensions are provided with accurate represent-tations | - Location, and scaling of Orthographic views is correct - Hidden features in Orthographic views are represented with $\leq3 errors$ - All Standard Dimensions are provided with $\leq$ 3 errors | - Location, and scaling of Orthographic views is correct - Hidden features in Orthographic views have $3-5 errors$ - Standard Dimensions have $3-5 errors$ | - Location, and scaling of Orthographic views is correct - Hidden features in Orthographic views have $>5 errors$ - Standard Dimensions have $>5 errors$ |
|  | | | | | |
| 3. Method of building bioreactor in CAD | **3 a. Method details and clarity**  If part making description does not fit a page, attach an Appendix to the report with detailed steps in tutorial form and provide an overview here. | - All special details & steps for part - assembly - drawing process are bullet-listed. - Method is written as a tutorial in a clear manner so that a classmate can easily regenerate the part with CAD. | - All special details of each steps are bullet-listed. - Writing is such that a classmate can regenerate the design in CAD using most of your directions, without getting stuck. | - Major steps are bullet-listed. - Directions must be improved for clarity so the person regenerating design does not get stuck. | - Only some steps are bullet-listed. - Directions do not enable a person to regenerate your design in CAD. |
| 4. CAD-based Drawings using Solidworks | **4 a. Assembly drawing** with exploded view | The assembly is shown correctly in exploded view. All parts are visible and labeled. The exploded view clearly shows where a specific part is located. Part number corresponds to its drawing in (4b) | The assembly drawing is shown correctly in exploded view. All parts are visible and labeled. Part number corresponds to actual part in part drawing (4b). | The assembly drawing is shown in exploded view. Parts are visible and labeled. Part number corresponds to actual part in part drawing (4b). | The assembly drawing is not correctly shown in exploded view. Parts are not clearly visible and labeled. Part number may not correspond to actual part in part drawing (4b). |
|  | **4 b. Separate images of each part drawings** must be provided by teammates | - All part drawings are shown after the assembly view. Each individual part is shown in its orthographic + isometric views in third angle projection. - Hidden features are correctly labeled. - All views are correctly dimensioned and show the unit of measure (mm /inches). - Dimensions are clear, readable, and concise with no repeat information. | - All part drawings are shown after the assembly view. Each individual part is shown in its orthographic + isometric views in third angle projection. - Hidden features are correctly labeled. - Views are correctly dimensioned and show the unit of measure (mm /inches). - Dimensions are readable, concise with mostly no repeat information. | - All part drawings are shown after the assembly view. Each individual part is shown in its orthographic + isometric views in third angle projection. - Hidden features are correctly labeled. - Most of the part drawings are correctly dimensioned and show the unit of measure (mm /inches), but dimensions are not clear (difficult to read). | - Incorrect representations of drawings in their orthographic + isometric views in third angle projection. - Hidden features are not correctly labeled. - Half of submitted part drawings are not correctly dimensioned. - Dimensions may not be readable. Unit of measure may not be presented. |
|  | | | | | |
| 5. Reflection on project | 5 a. Write-up describes **all team member’s contribution and learning in own words** | Reflection clearly mentions each team member’s contributions to the team, and creatively describes each member’s learning through the project in their own words. | Reflection mentions all team members’ contributions to the team, and team member’s own learning through this project in their own words. | Reflection mentions all team members’ contributions to the team, but only some team member’s own learning through this project in their own words. | Reflection mentions all members’ contributions to the team, but lacks member’s own learning experience. |
|  | | | | | |
| 6. Writing quality | **6 a. Quality + citations** | Clearly written using correct grammar. References are in IEEE format. | Some ambiguity and some grammatical errors. References in IEEE format. | Poorly written. Poor grammar. References missing or not in proper format. | Not intelligible. |

| Supplementary Table S.4: Rubric for Peer Assessment of Hand-drawn Sketch of Bioreactor | | | | |
| --- | --- | --- | --- | --- |
| **Criteria / Level of mastery** | **Proficient**  **(9 pts)** | **Developing**  **(6 pts)** | **Under-achieved**  **(3 pts)** | **Incomplete**  **(0 pts)** |
| A. View and feature- location, size and scaling | - Location of views and features is correct - Scaling of view sizes and features is correct - Scaling of feature dimensions is correct in all views | - Location of views and features is correct - Scaling of view sizes is correct - Scaling of dimensions is wrong in one view. | - Location of views and features is correct, - Scaling of view sizes is correct, but feature sizes wrong - Scaling of dimensions is incorrect in more than one views. | - Location of views or features is incorrect and - Scaling of view and /or feature sizes is incorrect   **OR**   - Scaling of dimensions is incorrect in 2 views. |
| B. Hidden features | - All hidden features shown (1 pt) - Hidden features are without any of the 8 representational errors discussed in class. ( 8 X 1 = 8 pts) | - $\leq$ 3 errors | - $3-5$errors | - $\geq$ 5 errors |
| C. Dimensions | Dimensions are:   - Not redundant. - Complete. - Not crossing each other. - Grouped on a drawing - Not using object lines as extension lines for a dimension. - Staggered with text. - Kept off the view, unless necessary for clarity. - Using the X symbol to show repetitive features. - Slots, concentric circles and arcs are dimensioned correctly. | - $\leq$ 3 errors in the list of Proficient column | - $3-5$errors   in the list of Proficient column | - $\geq$ 5 errors in the list of Proficient column |

References

1. Choi, J.H., et al., *Adipogenic differentiation of human adipose-derived stem cells on 3D silk scaffolds*, in *Adipose-Derived Stem Cells*. 2011, Springer. p. 319-330.

2. Salehi-Nik, N., et al., *Engineering parameters in bioreactor’s design: a critical aspect in tissue engineering.* BioMed research international, 2013. **2013**.

3. Oakley, B., et al., *Turning student groups into effective teams.* Journal of student centered learning, 2004. **2**(1): p. 9-34.

4. Learning, I. *Understand IBM Design Thinking in 10 minutes*. 2016; Available from: <https://www.youtube.com/watch?v=psLjEBUOnVs&t=3s>.

5. *The Heart of Your Grant Proposal: How to develop a strong need statement*.

6. Foundation, N.S. *The 2010 User-Friendly Handbook for Project Evaluation* 2010; Available from: <https://www.purdue.edu/research/docs/pdf/2010NSFuser-friendlyhandbookforprojectevaluation.pdf>.

7. Learning, J., *Engineering Design Decision Matrix*. Jason.org.
